# Achieving Precision Medicine in Allergic Disease: Progress and Challenges

**DOI:** 10.3389/fimmu.2021.720746

**Published:** 2021-08-18

**Authors:** Steven P. Proper, Nurit P. Azouz, Tesfaye B. Mersha

**Affiliations:** ^1^Division of Allergy and Immunology, Cincinnati Children’s Hospital Medical Center, Cincinnati, OH, United States; ^2^Department of Pediatrics, University of Cincinnati College of Medicine, Cincinnati, OH, United States; ^3^Division of Asthma Research, Cincinnati Children’s Hospital Medical Center, Cincinnati, OH, United States

**Keywords:** precision medicine, allergic disease, atopic march, omics, endotypes, exposome, immunophenotyping, machine learning

## Abstract

Allergic diseases (atopic dermatitis, food allergy, eosinophilic esophagitis, asthma and allergic rhinitis), perhaps more than many other traditionally grouped disorders, share several overlapping inflammatory pathways and risk factors, though we are still beginning to understand how the relevant patient and environmental factors uniquely shape each disease. Precision medicine is the concept of applying multiple levels of patient-specific data to tailor diagnoses and available treatments to the individual; ideally, a patient receives the right intervention at the right time, in order to maximize effectiveness but minimize morbidity, mortality and cost. While precision medicine in allergy is in its infancy, the recent success of biologics, development of tools focused on large data set integration and improved sampling methods are encouraging and demonstrates the utility of refining our understanding of allergic endotypes to improve therapies. Some of the biggest challenges to achieving precision medicine in allergy are characterizing allergic endotypes, understanding allergic multimorbidity relationships, contextualizing the impact of environmental exposures (the “exposome”) and ancestry/genetic risks, achieving actionable multi-omics integration, and using this information to develop adequately powered patient cohorts and refined clinical trials. In this paper, we highlight several recently developed tools and methods showing promise to realize the aspirational potential of precision medicine in allergic disease. We also outline current challenges, including exposome sampling and building the “knowledge network” with multi-omics integration.

## Introduction

### Atopic Disease Overlap

Allergic or atopic conditions all share an underlying degree of inappropriate immunologic response to what should otherwise be benign exposures/stimuli. The most common of these allergic conditions are atopic dermatitis (AD), IgE-mediated food allergy (FA), eosinophilic esophagitis (EoE), asthma, allergic rhinitis (AR), drug allergy, contact dermatitis, and urticaria/angioedema. Collectively they comprise some of the most common chronic disorders in both childhood and adulthood, and account for significant healthcare utilization and economic burden (1, 2). There is a clear clinical and pathophysiological association between these allergic conditions as patients diagnosed with a single allergic disorder, or with significant family history of these disorders, are at variably increased risk of being diagnosed with other allergic conditions (3). For example, a systematic review by van der Hulst et al. describes a pooled odds ratio for the risk of asthma in children with eczema compared to children without eczema of 2.14 (95% CI, 1.67-2.75) and approximately 1 in 3 children with eczema developing asthma during later childhood (4). Population-wide incidence of each condition varies by age, in what is often referred to as the “atopic march” (5). Many efforts in allergy research are currently aimed at unraveling the associations between these allergic diseases and understanding the various risk factors for allergic multimorbidity. Allergic conditions are influenced by variable factors such as diet, infections, exposure to antibiotics and chemicals, microbiome composition, and genetic and epigenetic elements which ultimately affect multiple molecular pathways (6). The confluence of these factors introduces significant heterogeneity to the molecular underpinnings, presentation, course and response to various treatments of each allergic disease. Defining the specific molecular mechanisms responsible for each individual disease variant or endotype, is a highly active area of allergy and immunology research (7) and is of great importance for developing tailored therapies.

### What’s in an Endotype?

**Figure 1:**
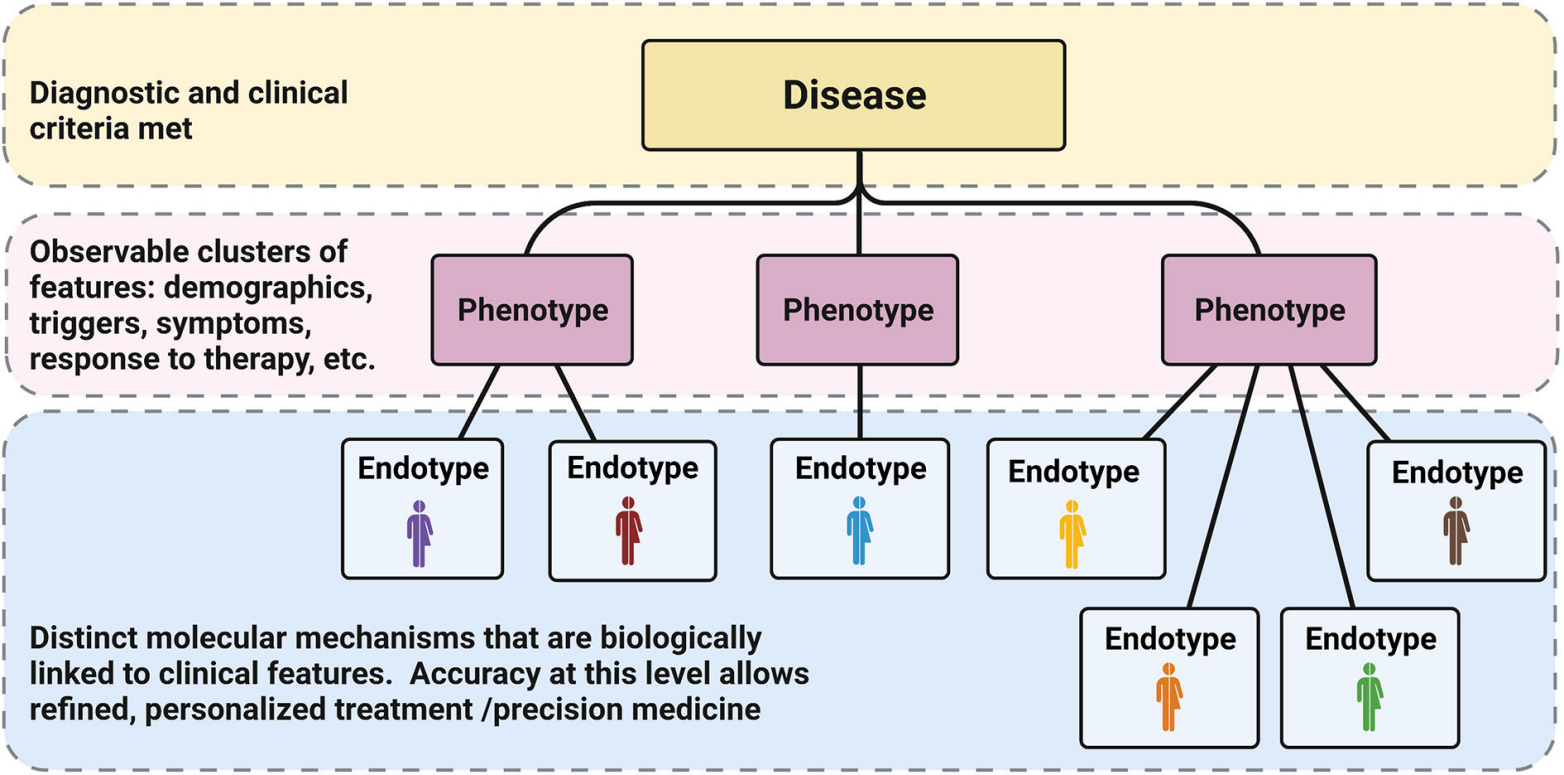
Phenotypes *vs* Endotypes: Classically diagnosis at the disease level (yellow) depends on meeting specific clinical criteria (usually linked to a specific dysfunction or feature). Diseases have been subdivided over time into phenotypes based on other observable characteristics and patterns (pink). In the current age of refined molecular analysis and big data, we have the opportunity to further characterize specific molecular markers that are biologically linked to the levels above, defining unique endotypes (blue). The accuracy of these distinctions will allow for specific, targeted therapy and truly allow for precision medicine. Reproduced from (8).

Classically, disease phenotypes are defined by some observable disease trait, such as demographic factors (age, sex), race, triggers, severity, response to certain treatments, etc. Disease endotypes differ in that they refer to specific molecular pathophysiologic mechanisms driving the disease in question. Endotypes are not necessarily limited to a single molecular marker, but rather represent the unique constellation of factors mechanistically responsible for the phenotype/trait? (**Figure 1**). In clinical practice our definition of allergic endotypes has been simplistic and more akin to molecular phenotypes than the idealized definition of endotype (i.e. high levels of eosinophil versus low in asthma or allergic asthma/IgE sensitization); nevertheless, these definitions have proven useful with the efficacy of biologics targeting these pathways. For example, patients with asthma and high sputum eosinophilia or glucocorticoid-dependence respond well to antibodies directed against IL-5 compared with asthmatics without these features. Similarly, asthmatics with IgE sensitization respond to IgE-directed therapy of omalizumab by decreased exacerbation rates and decreased seasonal peaks of symptoms (9, 10). These advances are welcome for both clinicians and patients alike, though they represent only a fractional improvement. Further clarification of the landscape of allergic endotypes is needed to reveal the potential of additional (or combinations of) directed therapies. Thus, it is important to think of most current endotypes as more akin to molecular phenotypes, since we are still mapping them with an incomplete taxonomy (8, 11). The presence of distinct molecular phenotypes is consistent with, but does not sufficiently define, distinct endotypes. Through advancing our understanding of the underlying mechanisms of disease, further resolution of atopic endotypes is possible.

### Precision Medicine

**Figure 2:**
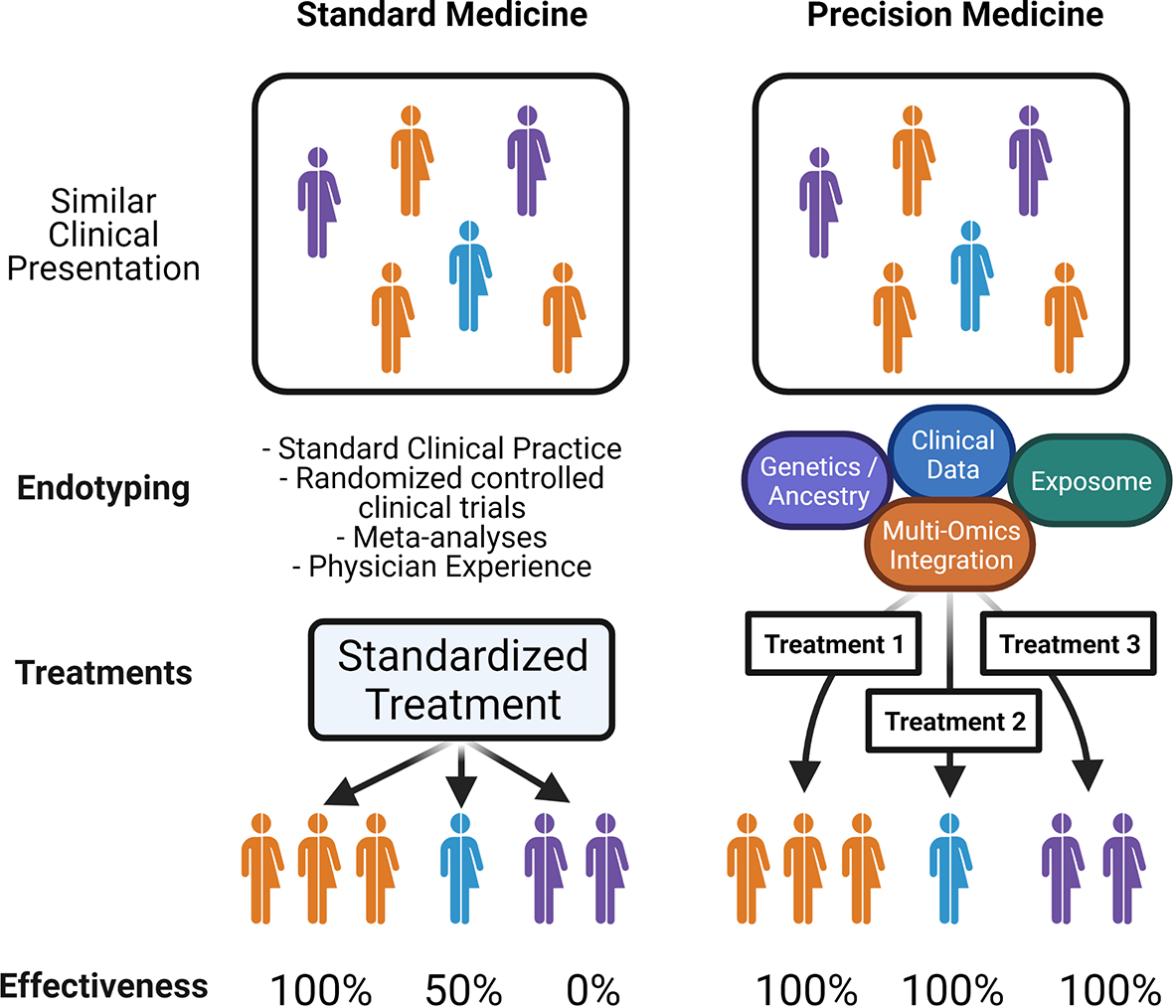
Precision Medicine in Allergy: Schematic illustrates; on the left, standard medical practice relies on clinical presentation or phenotypes, leading to limited endotypic classification, and standardized therapy which may be effective for only a subset of patients. On the right, an idealized precision medicine approach which takes into account all patient-specific data that reveals endotypes with common molecular pathways, in order to match patients with tailored treatments that have increased effectiveness.

Precision medicine (PM), sometimes in the past referred to as personalized medicine or P4 medicine [personalized, predictive, preventive, and participatory (12)], is a concept where individual patient data can be applied in such a way as to ensure that only the treatments that are most likely to be effective for that particular individual will be used – so they receive the right intervention at the right time, to maximize treatment effectiveness and minimize risks, morbidity, mortality, and cost (13). This concept emerged in the late 2000s in the aftermath the Human Genome Project and the advancement of next-generation sequencing along with other technologies that rapidly increased the amount of genetic data available for the study of disease. In 2011, the U.S. National Research Council published a report called “Toward Precision Medicine: Building a Knowledge Network for Biomedical Research and a New Taxonomy of Diseases” which further popularized the idea of precision medicine and a “Knowledge Network” of several data streams to drive further understanding of disease (14). The challenge in achieving this aspirational concept of precision medicine is determining which individual features are the most relevant for any particular disease context. Formalizing this disease context is what is meant by the idea of defining disease endotypes: taking a clinical disease and dividing it into specific subtypes of disease based on distinct pathologic and molecular mechanisms, rather than a classical constellation of clinical findings. If a disease endotype can be further refined, then individual patients could receive more endotype-specific diagnoses and thus optimized endotype-specific treatments, increasing the overall effectiveness of therapy (**Figure 2**). In order for precision medicine to realize its potential for allergic diseases, we will need a much greater understanding of the landscape of allergic endotypes.

### Precision Treatment in Allergy

While allergists have for several decades participated in the precision afforded by personalized allergen immunotherapy for allergic rhinitis and conjunctivitis (allergy shots), it was not until relatively recently that biologics offered more specific treatment options for other allergic diseases. The use of anti-interleukin (IL)-5 agents in asthma is an illustrative example of the power and simultaneous challenge of precision medicine in allergy (15). When anti-IL-5 antibodies were first tested in mild or moderate severity asthmatics, they failed to show clinical benefit despite reductions in eosinophils (16, 17); however, when studies were limited to steroid-dependent asthmatics with persistently elevated eosinophil counts, it performed well with significant steroid-sparing effect leading to the first new class of asthma medications in 12 years (18, 19). Subsequently, many of the Type 2 cytokines and others have been targeted in this fashion, and these advancements are allowing allergists and others to offer new treatments for the first time in many years (20). While it is true that these represent an exceptional advancement in our ability to treat those with most severe allergic conditions, it is clear that more work remains. Many additional candidate targets remain to be tested, and there is still a need to broaden the list of allergic biomarkers at our disposal. A recent review by Breiteneder et al. summarizes many of the latest efforts in identifying biomarkers in allergic disease (21).

Another advance in precision of allergy diagnostics is use of allergen component testing (referred to often as component-resolved diagnostics or CRD). Compared to classical testing of IgE to whole allergen extracts, CRD identifies binding to specific allergen proteins (either purified from extract or recombinant) and can aid in diagnosis and treatment (22). To date these have been most widely applied in the area of food allergy (23). For example, in the case of peanut allergy, specific IgE to Ara h 2 (2S albumin) is more associated with true peanut allergy and anaphylactic reactions compared to Ara h 8 and Ara h 9 which are typically not (24). CRD is generally available in the United States and elsewhere. Some diagnostic modalities combine CRD and extract testing in the forms of ImmunoCAP^®^ Solid-phase Allergen Chip (ISAC) multiplex testing and ALEX^®^ Allergy Explorer (most widely used in Europe) which utilizes microarray technology to perform multiple simultaneous analyses (25, 26). Although much more research is underway to determine clinical utility of CRD in other foods and allergic disease contexts, it is a promising advance with potential to enhance precision medicine in allergy.

It is also worth noting that sublingual immunotherapy (SLIT) and oral immunotherapy (OIT) have been significantly investigated and advanced in the last decade, offering some additional therapeutic options for patients (where available) and researchers alike (as additional trials are conducted). For SLIT, the only products that have been FDA approved in the US are tablet forms, notably for grass (Grastek^®^ and Oralair^®^), dust mite (Odactra^®^), and ragweed (Ragwitek^®^). Other liquid forms of SLIT are more widespread in Europe, and several studies have shown that both forms have demonstrated efficacy, though their clinical use varies (27–29). OIT had its first product for peanut allergy approved in the US (Palforzia^®^) in January 2020, building on several studies over the prior decade. Compared to avoidance, OIT subjects had much higher rates of anaphylaxis during treatment but also had increased rates of subsequent non-reactive peanut challenge (30). While OIT is thought of as essentially a prolonged desensitization, research is ongoing to understand whether sustained unresponsiveness (tolerance) can be achieved and the limits of OIT outcomes. Together, SLIT and OIT make use of the mucosal gastrointestinal epithelium to promote tolerance, and there are likely several endotypes of allergic disease for which these therapies may become part of optimal treatment.

Now that several new therapies are available for allergic conditions, even more study of patient responses, outcomes, phenotypes, and endotypes of allergic disease are possible and needed. Understanding these allergic endotypes is critical for advancing allergy treatment toward precision medicine. Two of the greatest challenges in endotyping allergic diseases are 1) data collection/sampling and 2) our ability to integrate and interpret a massive and growing amount of patient data. Advancing these factors will require tailored technologies, skill sets and tools. We will next review some recent examples of advancements to both sampling techniques and data integration tools specific to atopic disease and discuss how they can be leveraged to advance precision medicine in allergy and immunology.

## Advancements in Sampling

### Skin Tape Stripping

While the skin is one of the most readily accessible organs, obtaining biopsies (especially from younger patients) for research or even clinical diagnostics is challenging. One relatively recent advance gaining acceptance is skin tape stripping (STS) whereby layers of skin microbiota and *stratum corneum* are sampled by applying successive tape strips that are removed and subsequently analyzed. Other than some minor site irritation, these processes have relatively few side effects and are well tolerated by most subjects. Samples from STS are more superficial compared with skin biopsy and thus less disfiguring. Many groups are refining the process to maximize its clinical and research utility. For example, Leung et al. were able to show differences in trans-epidermal water loss (TEWL), filaggrin breakdown products, ceramides, and microbiome between AD patients with food allergy (peanut), AD patients without food allergy, and normal controls (31). They further demonstrated the ability to resolve differences in proteomic data using this STS system and cohort (32). Other groups have reported results of STS revealing differences in immune biomarkers in AD patients (33). While some STS studies have required pooling of tape strips to obtain good signal, Stevens et al. have demonstrated performance of single tapes showing changes that also differ with depth of sampling (number of tapes), and that performance of different taping systems varies widely (34). A recent review by Hughes et al. summarizes some of the recent progress made in STS and which samples are readily obtainable (35). Advancement of this technology may improve the consistency and yield of many kinds of biomarkers from skin tape stripping, and while it cannot replace the gold standard of skin biopsy, it is likely that this technology will remain in some form as a mainstay of dermatologic sampling.

### Suction Blister Formation

One of the more difficult cell types to sample and evaluate within allergic lesions are lymphocytes, dendritic cells, and other immune cells, due to their lower abundance compared to target organ cells. Obtaining immune cells from the organ of interest has a higher likelihood of identifying pathogenic cell populations than obtaining them from peripheral blood. Sampling of lung and gut epithelium is restricted to bronchoscopic and endoscopic procedures due to practical considerations, while skin is more accessible. Tape stripping has shown success with upper layers of skin such as the *stratum corneum*, but immune cells are seldom recovered from STS. In contrast, skin biopsies obtain high numbers of cells, but are quite invasive and scar-forming, and require tissue processing. One method less invasive than biopsy showing promise in immunophenotyping of skin is creation of suction (negative pressure) blisters and sampling the resulting blister fluid and the blister roof of epidermal cells (36). This technique is not scar-forming, and allows for cell isolation from blister roof and fluid as well as proteomic profiling (37).

One recent study by Rojahn et al. compared suction blister fluid (SBF) to skin biopsy in terms of proteomic and single-cell RNA-seq transcriptional analysis in AD patients compared to healthy controls, and revealed that SBF sampling was quite comparable to biopsy in terms of cell populations identified. SBF detected mRNAs from fewer cells, and some populations were not observed such as mast cells and non-migratory CD163+ macrophages; however, SBF had higher scRNA-seq specificity for high abundance transcripts (38). In another study by Sjöbom et al. comparing plasma and SBF, 70 proteins were detected in both plasma and SBF, with 38 out of 70 proteins enriched in SBF compared to 24 proteins being enriched in plasma. Of the 70 proteins shared between sample types, protein levels were significantly correlated in 25 (39). Together, these studies suggest that SBF can still identify many key target cell populations and offer unique proteomic sampling that is worth consideration in future studies. Willingness of patients to allow blister collection, in addition to the ability to perform simultaneous high fidelity proteomic and cell-specific transcriptional data, may give suction blister methods an advantage especially in cohorts of younger patients. Standardization of immune cell enrichment processes in blister fluid would be useful as this technology advances, though it is clear that suction blistering could provide another avenue for sampling the skin beyond the *stratum corneum*, and will be useful in endotyping atopic skin conditions.

### Mass Cytometry (CyTOF)

One of the most commonly used methods in immunology to clarify lymphocyte and other cell populations by surface markers or intracellular staining is flow cytometry, which has been limited in the total number of markers that could be simultaneously assayed. There are several emerging technologies aiming to improve the resolution of current immunophenotyping beyond the traditional 11-20 or so markers typical of flow cytometry (40). Multiple laser systems and use of spectral flow cytometry can improve this to 40 or so parameters, though these are much less common and there is extensive spectral overlap across probes to contend with (41, 42). One technology gaining popularity due to bypassing the need for fluorescent detection is mass cytometry with time-of-flight (CyTOF), which uses cell labeling with antibodies conjugated with unique isotopes of heavy metals and mass spectroscopy to detect labeling of each marker. The lanthanide series of metals has 35 isotopes in use, with several others in development (43). Some limitations of CyTOF are that processing of cells is somewhat slower, and cells are destroyed in the process of mass spectroscopy; however, the ability to reach nearly 50 markers makes this system an excellent option in assessing complex samples, and greatly improves immunophenotyping resolution.

Several recent studies in allergy have utilized CyTOF technology to gain insight into immune cell populations. In a study focused on subjects with red meat allergy (galactose-α-1,3-galactose, or α-gal) Cox et al. used CyTOF to enumerate B cell phenotypes, describing clusters of B cells with CD27^lo^, higher IgD, lower IgM and associated with CXCR4, CCR6 and CD25 expression that demonstrated secretion of alpha-gal specific IgE on stimulation (44). Whether these represent a naïve B cell population or memory B cells that lost CD27 expression is unclear, but this was the first study of its kind to immunophenotype B cells in alpha-gal to this degree. Classic skin prick testing to red meat in patients with α-gal has not been reliably positive, and while intradermal testing is more reliable, not all clinics perform intradermal testing. In addition, IgE levels to α-gal can be very low in some patients who meet clinical criteria (45). Due to delay in onset of symptoms, α-gal is a clinically difficult-to-identify disorder. CyTOF may provide avenues for much-needed diagnostics in this disorder (46).

In peanut allergy, Neeland et al. utilized CyTOF in 1-year olds to define lymphocyte populations among those with clinical peanut allergy compared to those who were sensitized but tolerant to peanut, showing that peanut allergic infants had increased populations of CD19^hi^HLADR^hi^ B cells, overproduction of TNFα, and increased frequency of peanut-specific CD4 T cells, whereas the peanut-sensitized but tolerant infants had hyper-responsive naïve CD4 T cells and increased plasmacytoid dendritic cells (47). Whether this will translate to a clinically useful assay is unclear, but identifiable differences in lymphocytes between tolerant and allergic patients hold promise to assist in sorting out patients who need to avoid peanut vs proceed to oral peanut challenge.

There has been a well-recognized variability in sputum of asthmatics, due to several factors (48). In an effort to better characterize immunophenotypes in sputum of asthma patients, Stewart et al. used CyTOF to analyze 42 markers in induced sputum from adult asthmatics compared to healthy controls (49). They found increased neutrophils and decreased macrophages in asthmatics, and further identified several unique clusters of these cells. Specifically, when focused on CD66b^+^ CD15^+^ granulocytes (the most abundant granulocyte population in these samples), asthmatics had 2 unique clusters of eosinophils that expressed IL-13 and CD69/IL-5 as well as neutrophils expressing IL-4Ra, IL-7Ra and Eotaxin. When clustering the second most abundant cell types, monocytes and macrophages (CD66b^-^/CD15^-^), asthmatics had higher abundance of macrophages with CD16^+^/CD69^+^/IL-17A^+^/Eotaxin^+^/IL-13^+^/IL-5^+^ as well as CD16^+^/CD69^+^ and CD16^+^/IL-17A^+^/IL-6^+^ macrophage clusters. This level of specificity to immunophenotypes in sputum of asthma patients was not so easily achieved previously, and the ability to obtain more detailed information about sputum leukocytes has potential for further subphenotyping and endotyping of asthma.

In an intriguing study of food protein-induced enterocolitis syndrome (FPIES) by Goswami et al., a combined investigation of antigen-responsive cells by flow cytometry, whole blood CyTOF and whole blood RNA sequencing before and after food challenge in patients with FPIES identifying systemic innate immune activation along with absence of B- or T-cell activation (50). These results were in agreement with another study by Mehr et al. looking at bulk transcriptional expression profiles in peripheral blood during FPIES reactions in infants which highlighted expression of TNF, CSF2, LPS, IL-1β and others common in the innate immune response (51). This holds significant promise for intervention targeting the innate immune system for patients with FPIES, and helps target further research into this disorder (52). Taken together, the studies listed above are by no means definitive, but offer unprecedented detail into immunophenotyping of allergic disease through use of CyTOF. Use of technologies like CyTOF to enumerate immune populations in larger studies are critical adjuncts to advancing our understanding of endotype-specific pathogenesis and development of specific therapies.

### Single-Cell RNA Sequencing (scRNA-seq)

The ability to resolve transcriptional profiles of the entire transcriptome to the individual cell level is one of the most exciting advances in gene expression analysis to date. The technology depends on placing millions of uniquely coded primers (allowing unique cell identification) on individual beads and pairing each bead to single cells using a flow cell, and next generation sequencing to enumerate transcripts (53). Cell populations can be differentiated according to expression of unique transcripts, and differences in cell populations and their transcription across disease states can be compared. As expected, cells and transcripts of high abundance are favored, and intact single cells require tissue processing, but even small numbers of cells can have their entire transcriptome assayed. Several recent studies have started to take advantage of this level of resolution for allergic conditions.

He et al. used scRNA-seq to compare AD subjects (both lesional and non-lesional skin) to healthy controls and identified increased Th2 and Th22 cells, CD1A^+^FCER1A^+^ inflammatory dendritic cells (DCs), a novel *COL6A5^+^COL18A1^+^* fibroblast population and a LAMP3+ DC population. The novel *COL6A5^+^COL18A1^+^* fibroblast population seemed mechanistically linked to the novel *LAMP3^+^* DC population, with fibroblast expression of *CCL2* and *CCL19*, and the DC population expressing the *CCL19 receptor CCR7*. Together these data indicate that the fibroblast population may play an active role in signaling to immune cells in lesional AD (54). Rojahn et al. saw similar Th2 and Th22 profiles in their scRNA-seq analysis comparing biopsies to suction blister analysis, but also noted enrichment for myeloid cells. Notably, the proteomic analysis agreed with the cellular analysis in that DC (CLEC7A, amphiregulin/AREG, EREG) and macrophage (CCL13) products were among top upregulated proteins (38). Given the various AD endotypes previously proposed based on differential involvement of various T-helper subtypes, this cell-specific analysis is certain to reveal more insights into critical cell populations in AD (55). Studies to date of AD using scRNA-seq still use a very limited number of patients due to complexity and cost of analysis, though larger studies will certainly be ongoing and will pave the way for optimizing the data pipeline for analyses of scRNA-seq in skin samples.

Another atopic condition utilizing scRNA-seq is eosinophilic esophagitis (EoE). Patients with EoE often undergo regular endoscopic evaluation throughout the process of managing EoE, and thus tissue samples have been captured and analyzed. Wen et al. have described two distinct T cell subsets in active EoE, one which constitutes an active Th2-like (GATA3^+^HPGDS^+^CRTH2^+^IL-17RB^+^FFAR3^+^CD4^+^) cell with high levels of IL-5 and IL-13 production, and a Treg-like (FOXP3^+^CD4^+^MAF^+^CTLA4^+^IL-10^+^) cell (56). Another scRNA-seq experiment in human esophageal tissue revealed 14 epithelial populations with distinct expression of kallikrein 5 and the proteinase-activated receptor (PAR)2 in EoE patients compared to control patients (57). Bulk RNA sequencing of esophageal biopsies in EoE has been performed more than single cell RNA sequencing, and has revealed important information about the molecular signatures of EoE (58). Ruffner et al. recently compared RNA sequencing of esophageal biopsy to blood in adults and children with and without EoE, and identified a strong IFN signature in EoE biopsies that was not found in the blood or in GERD subjects (59). As with studies in AD, scRNA-seq for EoE were performed on smaller cohorts of subjects, and as the technology scales to include a wider spectrum of disease, cell-specific transcription may provide essential information in further endotyping these atopic conditions.

### Proteomics Using DNA Probes

Proteomics has always lagged somewhat behind the capabilities of next-generation sequencing (NGS) for genetic and gene expression analysis due to the complexity of the proteome, but the proteome arguably represents the most functional level of information and thus most significant opportunity for endotyping and biomarker identification. Some of the biggest challenges in proteomics involve adequate detection across a wide dynamic range of multiple proteins in a single sample. ELISAs require large volumes of sample, and even antibody-based multiplex assays have limits to the number of proteins/markers per sample and still suffer from sensitivity issues and batch effects. Recent developments that have advanced this field dramatically include technologies that utilize DNA probes paired with antibodies (Proximity Extension Assay [PEA], e.g. Olink technology) or DNA probes which are themselves modified for specific protein binding (Aptamer arrays, e.g. SOMAscan technology), which are reviewed expertly by Smith and Gerszten (60). Protein specificity combined with PCR and NGS technology dramatically increase the sensitivity and scalability of proteomics in biological samples and have already begun to have an impact.

In atopic dermatitis, Pavel et al. compared the proteome of blood to skin biopsies in adult subjects with AD (lesional and non-lesional) and healthy controls using the Olink PEA platform, and also compared this to bulk RNA sequencing of the same samples (61). The proteomic platform identified 354 proteins and was the largest proteomic profile of AD at the time. They showed an increase in protein of inflammatory markers in skin compared to blood, demonstrating proof-of-concept for utilizing Olink PEA for robust skin proteomics using as little as 10 µg of sample protein, which is obtainable in a 1 mm punch biopsy or 10 µL serum. Expansion of this platform to childhood AD or use in prospective cohorts would be extremely useful to our understanding of AD phenotypes and time course. Similarly, Rojahn et al. utilized the Olink PEA system to quantify 368 proteins in suction blister fluid to complement their comparison of scRNA-seq between suction blister and biopsy, which allowed some degree of validation of changes in gene expression leading to changes in protein (38). Both of these studies validate the PEA platform and show its versatility both with biopsy and suction blister fluid.

Another platform with the ability to detect hundreds of proteins simultaneously, SOMAscan, was utilized by Leonard et al. to detect 1129 proteins in sera among 76 adult subjects with AD compared to healthy controls to develop a detailed proteome of AD serum. Uniquely, they also utilized the ImmunoCAP Solid-phase Allergen Chip (ISAC) to pair sensitization analysis to the proteome. As expected, IgE was elevated across all AD groups compared to controls, though each sensitization group displayed unique proteomic profiles (food, perennial, seasonal, mixed), with seasonal and perennial sensitization linked to increased IgE to toxic shock syndrome toxin-1 of *S.aureus* (62). The limitation of this study was the small group sizes among sensitization groups, but this approach holds promise to link specific proteomic profiles to specific sensitization patterns.

In a study comparing sputum between smokers and non-smokers with mild/moderate and severe asthma, Rossios et al. used SOMAscan assay to show the eosinophilic severe asthmatics (both smokers and non-smokers) had increased expression of IL-1 receptor family members, whereas neutrophilic severe asthmatics had increased inflammasome signaling *via* nucleotide-binding oligomerization domain, leucine-rich repeat and pyrin domain containing 3 (*NLRP3*) expression (63). Interestingly, there were many subjects with high eosinophil counts but little or no IL-13/Type 2 signatures as well as low eosinophil counts/higher neutrophils with high IL-13/Type 2 signatures, indicating a more complex relationship between sputum eosinophilia and IL-13/Type 2 signature. Also of note, changes in inflammasome activation were not observed in bronchial brushings or biopsies, suggesting that sputum may be a more ideal specimen type in some studies.

Even if proteomics are not resolved to the single cell level, the ability to simultaneously assay 300 or more proteins from such small samples is an incredible advance. Limits of these technologies are that they are biased (pre-selected targets), requiring development of specific probes per protein target, though these technologies appear to scale well with the addition of more probes. These techniques would pair well with other unbiased proteomic methods of detection for unforeseen targets for a more complete sampling. These new proteomics tools offer significant advances to sampling that will continue to have an impact in characterizing allergic molecular phenotypes as they are more widely utilized.

## Advances in Omics and Machine Learning

Recent advances in omics technologies have led to unprecedented efforts to molecularly characterize the development and progression of a wide array of common complex human diseases, including allergy. Multi-omics analyses take advantage of these technologies in genomics, transcriptomics, epigenomics, proteomics, metabolomics, and other omics areas to advance precision medicine. Although multi-omics data has the potential for disease prevention, early detection and monitoring progression, truly integrated multi-omics analyses have not been applied widely. Additional efforts are needed to develop the analytical methods, including machine learning, to analyze, annotate and integrate multi-omics data to inform precision medicine-based decision-making.

Machine Learning (ML) refers to a methodology in the domain of data analytics that automates systematic building of the model. It permits the discovery of unseen insights from enormous datasets by means of suitable methods which involve repetitive learning gathered from the data devoid of being programmed explicitly. Traditional machine learning algorithms such as Naive Bayes, ANN and k nearest neighbor (KNN) use a fixed mathematic formula to make predictions and have overfitting problems. Deep learning (DL) is a subset of ML algorithms characterized by the use of artificial neural networks (ANN) (64). ANNs are inspired by biological neural networks in the sense that they are formed by interconnected artificial neurons, which receive an input, apply a transformation to the data, and return an output. DL lies at the intersection of statistics and computer science and does not rely on fixed mathematic formulas and has more than 100 layers to teach itself. The deeper the layer, the better it can learn and the more accurate the prediction. Deep learning uses larger numbers of hidden layers whereas traditional ANNs normally can only afford one or two hidden layers. Deep learning methods have achieved considerable improvements in classical artificial intelligence challenges like language processing, speech recognition, and image recognition (65).

With the advances of the big data era in biology such as omics, data analysis is frequently impeded by low signal to noise ratios, with large number of variables and relatively small number of samples. DL algorithms not only analyze each omics type separately but also have the opportunity to integrate multi-omics layers (**Box 1**) including data from clinical or health records with great sensitivity, specificity and efficiency (66). As newer advances in sampling increase the depth and breadth of big data gathered, and as sample number increases in cohorts, DL has the potential to reveal many valuable insights we would otherwise be unable to discern with classical approaches.

**Box 1. Definitions of Terms**

**Omics:** Field in biology that investigates molecular information (e.g. genome, proteome) in a cell/tissue.
**Genetics:** refers to the study of inheritance and the ways that traits or conditions are passed down from one generation to another.
**Genome:** A comprehensive DNA sequence of an organism, including interactions of those genes with each other and with the person's environment.
**SNP:** Single nucleotide polymorphism (SNP) is a variation of a single nucleotide in a genome locus.
**Transcriptome:** A snapshot of all messenger RNA (mRNA) transcripts that can be found in a cell/tissue at a given time. The transcriptome echoes the dynamic environment inside the cell.
**Epigenome:** A complete set of nuclear information that is not coded in DNA and can affect gene expression. It includes DNA methylations, histones modifications and alterations in chromatin structure.
**Proteome:** All proteins and peptides identified in cells/tissues at a given time.
**Metabolome:** A snapshot of all low molecular weight metabolites—(small molecules such as amino acids, carbohydrates, etc.)—that can be identified in cells/tissue at a given time. Metabolites are products of metabolic processes.
**Microbiome:** Quantity and quality of other organisms (bacteria, fungi, viruses) identified in a given compartment/organ (e.g. gut, skin, airway). Metagenomics studies of the microbiome usually apply 16s RNA sequencing to infer genus/species.
**Exposome:** All internal and external conditions that cumulatively influence an organism throughout its life.
**GWAS:** Genome- Wide Association Studies aim to find associations between genome with phenotypes (disease, drug response, physiological characteristics).
**eQTL**: expression Quantitative Trait Loci refers to how specific genetic loci are associated with expression level and beyond (methylation, protein abundance or metabolites), so functional relevance of loci can be inferred.
**Genome-Wide Interaction Studies:** are used to infer interactions between some environmental exposure (e.g. microbial) and phenotypic characteristics.
**miRNA:** micro RNA is a type of noncoding RNA that may influence gene expression levels, for example by degrading mRNA.
**Histone modifications:** Histones are the proteins involved in DNA packing and may influence gene expression. Histones can be modified by methylation/ demethylation, acetylation/deacetylation, ubiquitination, etc.
**Systems biology:** A study of interactions in molecular pathways that is based on computational methods and mathematical models.
**Single‐cell Omics:** collected at a single-cell level.

## Challenges in Sampling – The Exposome

**Figure 3:**
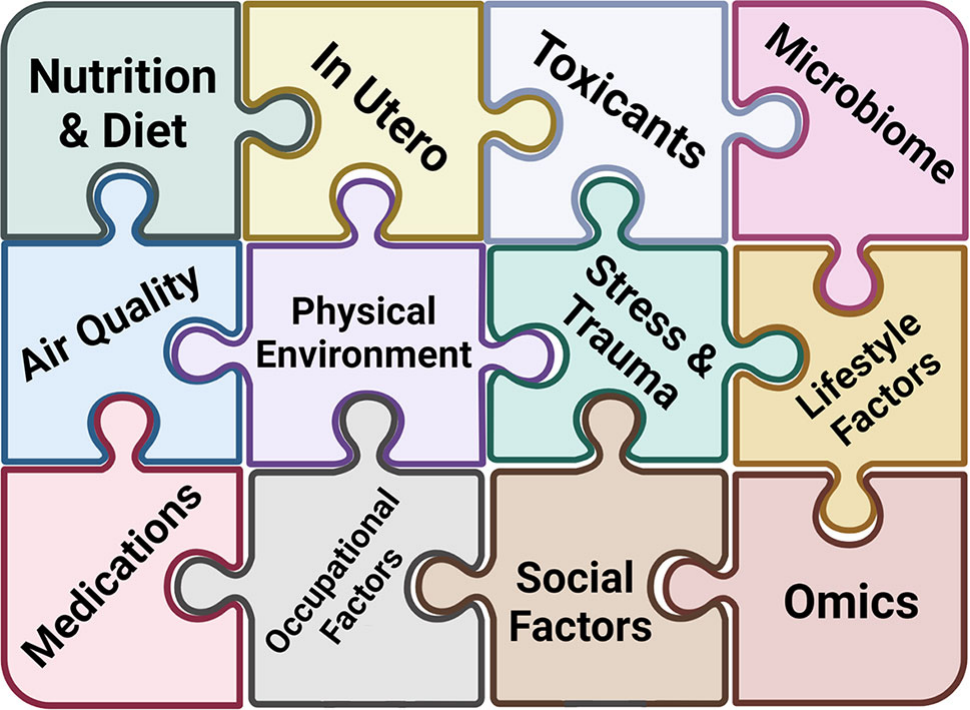
Understanding Complex Allergic Disease through the Exposome. The exposome encompasses the totality of human environmental exposures. Each piece of the exposome puzzle adds more information that has the potential to discern the pathogenesis of complex allergic diseases.

The concept of the exposome was introduced in 2005 by Dr. Christopher Wild and encompasses “all exposures from conception onwards” (67). A multitude of exposure categories can be described, some of which are shared (i.e. community air/water/climate), some which are more individualized and specific (i.e. food/medication/animal exposure), and some which are a combination of both (behavioral/social/cultural) (68–73) (**Figure 3**).

Since exposures are central to allergic disease, framing our understanding of allergic diseases in terms of the individual level exposome is likely to yield new insights. Indeed, many recent reviews have superbly outlined our understanding of the leading factors of the exposome in allergic disease (74–78). It seems that almost everywhere we look, we can see the impact of specific factors of the exposome on allergic disease. The common theme that emerges is the enormous complexity of possible exposures. This complexity represents two distinct challenges, for which innovations are needed: sampling multiple exposures simultaneously (ideally being both validated and practical) and analysis of these multiple exposures (statistical analysis of a high number of variables).

As we move to PM initiatives, data generated from personal devices such as wearables including activity, air pollution, temperature, and physiologic data identify environmental and biological triggers in real time have an immense potential to improve sampling and provide objective assessment of disease susceptibility and therapeutic outcomes (79). Currently, however, few environmental risk factors have been incorporated into larger cohorts and risk models, and wearables represent both a challenge and opportunity still under active development (80, 81). Our ability to incorporate the exposome into allergy & immunology research will be in lock step with our ability to scale robust sampling methods to assess mixed exposures and integrate these multiple variables into cohort studies.

In addition, a key element of precision medicine is also the accurate assessment and utilization of ancestry to understand its impact on disease susceptibility and therapies. However, population-based research has often relied on social constructs of race and ethnicity which are a poor proxy for ancestry (82).

## Challenges in Building the Knowledge Network for PM

While many newer technologies can reveal finer resolution than previously achieved, organizing these data into the wider “knowledge network” remains a challenge. For example, Raffield et al. review comparisons of proteomics across cohorts and platforms, and while generally all methods show reasonable correlations, there are several factors to consider when integrating these various methods of proteomic analysis (83). Similar challenges exist for gene expression, especially with scRNA-seq platforms and the various possible workflows for these data, reviewed recently by Leucken et al. (84). Integrating these new data sets into the knowledge network will likely require databases to include raw data so that the most up to date analytical pipelines can be applied iteratively as the network expands.

As rapid technological advances make it routine for genomics researchers worldwide to generate increasingly large, complex, and diverse datasets, the challenges of managing and leveraging to efficiently use such data also increase. In addition, several design and methodological challenges such as patient cohorts that are appropriately powered for the specific risk factors and questions being answered still need to be tackled before omics can be applied in patient care. Addressing these challenges require the collective cutting-edge expertise of computational, statistical, and genomic scientists along with domain knowledge researchers developing innovative approaches, methods, and technologies. Ultimately, such efforts provide greater access to secure data and computational tools that facilitate multi-omics studies that will facilitate the implementation of precision medicine in allergic disease.

## Conclusion

**Figure 4:**
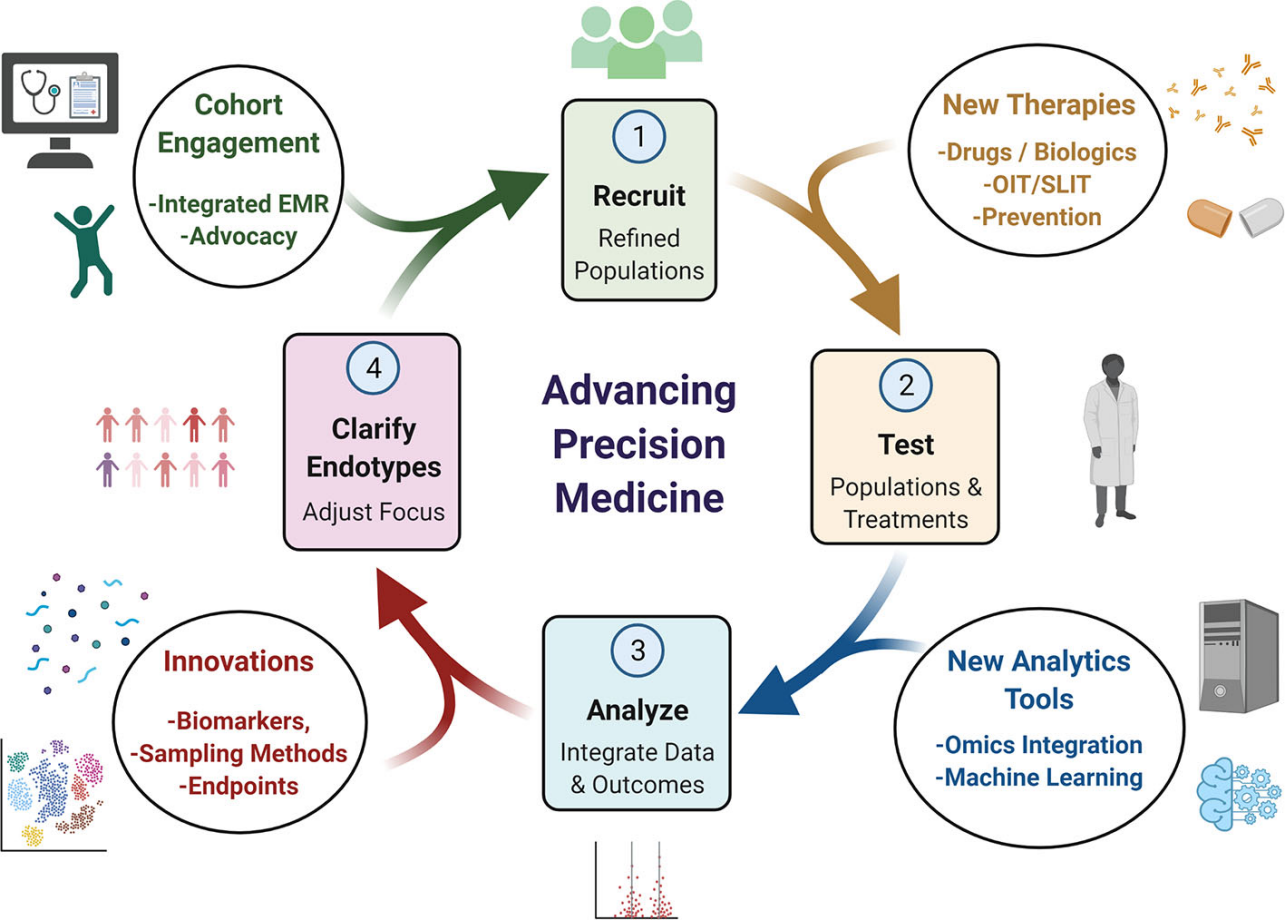
Conceptual Cycle of Precision Medicine Advancement. Advancement of precision medicine depends on an iterative process of clarifying endotypes, recruiting new cohorts designed to refine endotypes, which are then tested and analyzed. Innovations in biomarkers, improved sampling and new endpoints can offer new targets to study. Endotypes can be targeted more specifically for tailored cohort generation with next generation EMR systems and engaging with patient advocacy groups. New drugs or other therapeutic strategies can also be tested with these refined cohorts. Finally, integration of results and data tools can help refine the next iteration of which endotypes could be focused on. These innovations can impact any aspect.

Much like quality improvement (QI) in healthcare, precision medicine is more of an ideal and overarching process based on iteration rather than a specific destination. In order for the cycle of precision medicine to advance it needs to be fed by a robust basic and translational science infrastructure (**Figure 4**). New tools will broaden the power of existing data sets and lead to improved specificity in endotyping, so that focused, well-powered clinical trials can be conducted and therapies with higher precision can be implemented. We have outlined some examples in this brief review, but believe that the future is indeed bright for realizing the ideals of precision medicine in allergy & immunology.

## Data Availability Statement

The original contributions presented in the study are included in the article/supplementary material. Further inquiries can be directed to the corresponding author.

## Author Contributions

SP and TM conceptualized the topic and wrote the manuscript and NA contributed to write the manuscript. All authors contributed to the article and approved the submitted version.

## Funding

This work is supported by the National Heart, Lung, and Blood Institute (NHLBI) grant R01 HL132344 and the National Institute of Environmental and Health Sciences (NIEHS) grant T32 ES010957.

## Conflict of Interest

The authors declare that the research was conducted in the absence of any commercial or financial relationships that could be construed as a potential conflict of interest.

## Publisher’s Note

All claims expressed in this article are solely those of the authors and do not necessarily represent those of their affiliated organizations, or those of the publisher, the editors and the reviewers. Any product that may be evaluated in this article, or claim that may be made by its manufacturer, is not guaranteed or endorsed by the publisher.

## Abbreviations

AD, atopic dermatitis; α-gal, alpha-gal (galactose-α-1,3-galactose); AIT, allergen immunotherapy; ANN, artificial neural network; AR, allergic rhinitis; CyTOF, (mass) cytometry time of flight; CRD, component-resolved diagnostics; DC, dendritic cell; DL, deep learning; ELISA, enzyme-linked immunosorbent assay; EMR, electronic medical record (s); EoE, eosinophilic esophagitis; eQTL, expression quantitative trait loci; FA, food allergy; FPIES, food-protein-induced enterocolitis syndrome; GWAS, genome-wide association study/studies; IL, interleukin; KNN, k nearest neighbor; mRNA, messenger RNA; miRNA, micro RNA; ML, machine learning; NGS, next generation sequencing; OIT, oral immunotherapy; PEA, proximity extension assay; PM, precision medicine; QI, quality improvement; SBF, suction blister fluid; scRNA-seq, single cell RNA sequencing; SLIT, sublingual immunotherapy; STS, skin tape stripping; TEWL, trans-epithelial water loss; Th2, T-helper type 2.

## References

[1] CDC. Gateway to Health Communication and Social Marketing Practice - Allergies. Allergies [Online]. US Centers for Disease Control and Prevention. Available: http://www.cdc.gov/healthcommunication/ToolsTemplates/EntertainmentEd/Tips/Allergies.html [Accessed February 8th 2019].

[2] WAO. WAO White Book on Allergy: Update 2013. Milwaukee, WI, USA: World Allergy Organization (WAO) (2013).

[3] Celakovska J Bukac J. Analysis of Food Allergy in Atopic Dermatitis Patients - Association With Concomitant Allergic Diseases. Indian J Dermatol (2014) 59:445–50. 10.4103/0019-5154.139867 PMC4171910 25284847

[4] van der Hulst AE Klip H Brand PL. Risk of Developing Asthma in Young Children With Atopic Eczema: A Systematic Review. J Allergy Clin Immunol (2007) 120:565–9. 10.1016/j.jaci.2007.05.042 17655920

[5] Czarnowicki T Krueger JG Guttman-Yassky E. Novel Concepts of Prevention and Treatment of Atopic Dermatitis Through Barrier and Immune Manipulations With Implications for the Atopic March. J Allergy Clin Immunol (2017) 139:1723–34. 10.1016/j.jaci.2017.04.004 28583445

[6] Paller AS Spergel JM Mina-Osorio P Irvine AD. The Atopic March and Atopic Multimorbidity: Many Trajectories, Many Pathways. J Allergy Clin Immunol (2019) 143:46–55. 10.1016/j.jaci.2018.11.006 30458183

[7] Davidson WF Leung DYM Beck LA Berin CM Boguniewicz M Busse WW. Report From the National Institute of Allergy and Infectious Diseases Workshop on “Atopic Dermatitis and the Atopic March: Mechanisms and Interventions”. J Allergy Clin Immunol (2019) 143:894–913. 10.1016/j.jaci.2019.01.003 30639346 PMC6905466

[8] Fitzpatrick AM Bacharier LB. One Step Forward, 2 Steps Back: The Enigma of Preschool Wheeze. J Allergy Clin Immunol (2019) 143:1734–5. 10.1016/j.jaci.2019.01.005 30660640

[9] Normansell R Walker S Milan SJ Walters EH Nair P. Omalizumab for Asthma in Adults and Children. Cochrane Database Syst Rev (2014) CD003559. 10.1002/14651858.CD003559.pub4 24414989 PMC10981784

[10] Busse WW Morgan WJ Gergen PJ Mitchell HE Gern JE Liu AH. Randomized Trial of Omalizumab (Anti-IgE) for Asthma in Inner-City Children. N Engl J Med (2011) 364:1005–15. 10.1056/NEJMoa1009705 PMC3093964 21410369

[11] Shoda T Wen T Aceves SS Abonia JP Atkins D Bonis PA. Eosinophilic Oesophagitis Endotype Classification by Molecular, Clinical, and Histopathological Analyses: A Cross-Sectional Study. Lancet Gastroenterol Hepatol (2018) 3:477–88. 10.1016/S2468-1253(18)30096-7 PMC5997568 29730081

[12] Hood L Flores M. A Personal View on Systems Medicine and the Emergence of Proactive P4 Medicine: Predictive, Preventive, Personalized and Participatory. N Biotechnol (2012) 29:613–24. 10.1016/j.nbt.2012.03.004 22450380

[13] Mersha TB Hershey K Gurjit K. Precision Medicine in Allergic Disorders. In: Burks AW Holgate S T O’Hehir R E Bacharier LB Briode DH Khurana H Gurjit K Peebles RS, editors. Middleton’s Allergy: Principles and Practice, 9th ed, vol. 2. New York: Elsevier (2020). p. 1441–50.

[14] NRC. Toward Precision Medicine: Building a Knowledge Network for Biomedical Research and a New Taxonomy of Disease. Washington (DC): National Research Council (NRC), United States of America (2011). 22536618

[15] Mersha TB Afanador Y Johansson E Proper SP Bernstein JA Rothenberg ME. Resolving Clinical Phenotypes Into Endotypes in Allergy: Molecular and Omics Approaches. Clin Rev Allergy Immunol (2020) 60:200–19. 10.1007/s12016-020-08787-5 PMC8209667 32378146

[16] Leckie MJ ten Brinke A Khan J Diamant Z O’Connor BJ Walls CM. Effects of an Interleukin-5 Blocking Monoclonal Antibody on Eosinophils, Airway Hyper-Responsiveness, and the Late Asthmatic Response. Lancet (2000) 356:2144–8. 10.1016/S0140-6736(00)03496-6 11191542

[17] Flood-Page P Swenson C Faiferman I Matthews J Williams M Brannick L. A Study to Evaluate Safety and Efficacy of Mepolizumab in Patients With Moderate Persistent Asthma. Am J Respir Crit Care Med (2007) 176:1062–71. 10.1164/rccm.200701-085OC 17872493

[18] Nair P Pizzichini MM Kjarsgaard M Inman MD Efthimiadis A Pizzichini E. Mepolizumab for Prednisone-Dependent Asthma With Sputum Eosinophilia. N Engl J Med (2009) 360:985–93. 10.1056/NEJMoa0805435 19264687

[19] Rothenberg ME. Humanized Anti-IL-5 Antibody Therapy. Cell (2016) 165:509. 10.1016/j.cell.2016.04.020 27104969

[20] Pelaia C Crimi C Vatrella A Tinello C Terracciano R Pelaia G. Molecular Targets for Biological Therapies of Severe Asthma. Front Immunol (2020) 11:603312. 10.3389/fimmu.2020.603312 33329598 PMC7734054

[21] Breiteneder H Peng YQ Agache I Diamant Z Eiwegger T Fokkens WJ. Biomarkers for Diagnosis and Prediction of Therapy Responses in Allergic Diseases and Asthma. Allergy (2020) 75:3039–68. 10.1111/all.14582 PMC7756301 32893900

[22] Midun E Radulovic S Brough H Caubet JC. Recent Advances in the Management of Nut Allergy. World Allergy Organ J (2021) 14:100491. 10.1016/j.waojou.2020.100491 33510829 PMC7811165

[23] Flores Kim J McCleary N Nwaru BI Stoddart A Sheikh A. Diagnostic Accuracy, Risk Assessment, and Cost-Effectiveness of Component-Resolved Diagnostics for Food Allergy: A Systematic Review. Allergy (2018) 73:1609–21. 10.1111/all.13399 PMC6055682 29319184

[24] Nilsson C Berthold M Mascialino B Orme ME Sjolander S Hamilton RG. Accuracy of Component-Resolved Diagnostics in Peanut Allergy: Systematic Literature Review and Meta-Analysis. Pediatr Allergy Immunol (2020) 31:303–14. 10.1111/pai.13201 31872899

[25] Vankova R Celakovska J Bukac J Krcmova I Krejsek J Andrys C. Sensitization to Molecular Components in 104 Atopic Dermatitis Patients in Relation to Subgroups of Patients Suffering From Bronchial Asthma and Allergic Rhinitis. Acta Med (Hradec Kralove) (2020) 63:164–75. 10.14712/18059694.2020.59 33355077

[26] Čelakovská J Bukač J Cermákova E Vaňkova R Skalská H Krejsek J. Analysis of Results of Specific IgE in 100 Atopic Dermatitis Patients With the Use of Multiplex Examination ALEX2-Allergy Explorer. Int J Mol Sci (2021) 22:5286–317. 10.3390/ijms22105286 PMC8156228 34067936

[27] Field K Blaiss MS. Sublingual Versus Subcutaneous Immunotherapy for Allergic Rhinitis: What Are the Important Therapeutic and Real-World Considerations? Curr Allergy Asthma Rep (2020) 20:45. 10.1007/s11882-020-00934-4 32548677

[28] Dhami S Nurmatov U Arasi S Khan T Asaria M Zaman H. Allergen Immunotherapy for Allergic Rhinoconjunctivitis: A Systematic Review and Meta-Analysis. Allergy (2017) 72:1597–631. 10.1111/all.13201 28493631

[29] Roberts G Pfaar O Akdis CA Ansotegui IJ Durham SR Gerth van Wijk R. EAACI Guidelines on Allergen Immunotherapy: Allergic Rhinoconjunctivitis. Allergy (2018) 73:765–98. 10.1111/all.13317 28940458

[30] Chu DK Wood RA French S Fiocchi A Jordana M Waserman S. Oral Immunotherapy for Peanut Allergy (PACE): A Systematic Review and Meta-Analysis of Efficacy and Safety. Lancet (2019) 393:2222–32. 10.1016/S0140-6736(19)30420-9 31030987

[31] Leung DYM Calatroni A Zaramela LS LeBeau PK Dyjack N Brar K. The Nonlesional Skin Surface Distinguishes Atopic Dermatitis With Food Allergy as a Unique Endotype. Sci Transl Med (2019) 11:2685–97. 10.1126/scitranslmed.aav2685 PMC7676854 30787169

[32] Goleva E Calatroni A LeBeau P Berdyshev E Taylor P Kreimer S. Skin Tape Proteomics Identifies Pathways Associated With Transepidermal Water Loss and Allergen Polysensitization in Atopic Dermatitis. J Allergy Clin Immunol (2020) 146:1367–78. 10.1016/j.jaci.2020.04.022 PMC7606732 32360271

[33] Guttman-Yassky E Diaz A Pavel AB Fernandes M Lefferdink R Erickson T. Use of Tape Strips to Detect Immune and Barrier Abnormalities in the Skin of Children With Early-Onset Atopic Dermatitis. JAMA Dermatol (2019) 155:1358–70. 10.1001/jamadermatol.2019.2983 PMC6802262 31596431

[34] Stevens ML Gonzalez T Schauberger E Baatyrbek Kyzy A Andersen H Spagna D. Simultaneous Skin Biome and Keratinocyte Genomic Capture Reveals Microbiome Differences by Depth of Sampling. J Allergy Clin Immunol (2020) 146:1442–5. 10.1016/j.jaci.2020.04.004 PMC7572476 32320735

[35] Hughes AJ Tawfik SS Baruah KP O’Toole EA O’Shaughnessy RFL. Tape Strips in Dermatology Research. Br J Dermatol (2021) 185:26–35. 10.1111/bjd.19760 33370449

[36] Chen YL Gutowska-Owsiak D Hardman CS Westmoreland M MacKenzie T Cifuentes L. Proof-Of-Concept Clinical Trial of Etokimab Shows a Key Role for IL-33 in Atopic Dermatitis Pathogenesis. Sci Transl Med (2019) 11:2945–55. 10.1126/scitranslmed.aax2945 31645451

[37] Holm LL Vukmanovic-Stejic M Blauenfeldt T Benfield T Andersen P Akbar AN. A Suction Blister Protocol to Study Human T-Cell Recall Responses In Vivo. J Vis Exp (2018) 138:57544. 10.3791/57554 PMC6126709 30148487

[38] Rojahn TB Vorstandlechner V Krausgruber T Bauer WM Alkon N Bangert C. Single-Cell Transcriptomics Combined With Interstitial Fluid Proteomics Defines Cell Type-Specific Immune Regulation in Atopic Dermatitis. J Allergy Clin Immunol (2020) 146:1056–69. 10.1016/j.jaci.2020.03.041 32344053

[39] Sjobom U Christenson K Hellstrom A Nilsson AK. Inflammatory Markers in Suction Blister Fluid: A Comparative Study Between Interstitial Fluid and Plasma. Front Immunol (2020) 11:597632. 10.3389/fimmu.2020.597632 33224151 PMC7670055

[40] McKinnon KM. Flow Cytometry: An Overview. Curr Protoc Immunol (2018) 120:5.1.1–5.1.11. 10.1002/cpim.40 29512141 PMC5939936

[41] Jiang J Li X Mao F Wu X Chen Y. Small Molecular Fluorescence Dyes for Immuno Cell Analysis. Anal Biochem (2021) 614:114063. 10.1016/j.ab.2020.114063 33306976 PMC8043801

[42] Nolan JP Condello D. Spectral Flow Cytometry. Curr Protoc Cytom (2013) 63:1271–3. 10.1002/0471142956.cy0127s63 PMC3556726 23292705

[43] Han G Spitzer MH Bendall SC Fantl WJ Nolan GP. Metal-Isotope-Tagged Monoclonal Antibodies for High-Dimensional Mass Cytometry. Nat Protoc (2018) 13:2121–48. 10.1038/s41596-018-0016-7 PMC7075473 30258176

[44] Cox KM Commins SP Capaldo BJ Workman LJ Platts-Mills TAE Amir ED. An Integrated Framework Using High-Dimensional Mass Cytometry and Fluorescent Flow Cytometry Identifies Discrete B Cell Subsets in Patients With Red Meat Allergy. Clin Exp Allergy (2019) 49:615–25. 10.1111/cea.13322 PMC6488422 30506749

[45] Commins SP Satinover SM Hosen J Mozena J Borish L Lewis BD. Delayed Anaphylaxis, Angioedema, or Urticaria After Consumption of Red Meat in Patients With IgE Antibodies Specific for Galactose-Alpha-1,3-Galactose. J Allergy Clin Immunol (2009) 123:426–33. 10.1016/j.jaci.2008.10.052 PMC3324851 19070355

[46] Platts-Mills TAE Li RC Keshavarz B Smith AR Wilson JM. Diagnosis and Management of Patients With the Alpha-Gal Syndrome. J Allergy Clin Immunol Pract (2020) 8:15–23.e1. 10.1016/j.jaip.2019.09.017 31568928 PMC6980324

[47] Neeland MR Andorf S Manohar M Dunham D Lyu SC Dang TD. Mass Cytometry Reveals Cellular Fingerprint Associated With IgE+ Peanut Tolerance and Allergy in Early Life. Nat Commun (2020) 11:1091. 10.1038/s41467-020-14919-4 32107388 PMC7046671

[48] Simpson JL Scott R Boyle MJ Gibson PG. Inflammatory Subtypes in Asthma: Assessment and Identification Using Induced Sputum. Respirology (2006) 11:54–61. 10.1111/j.1440-1843.2006.00784.x 16423202

[49] Stewart E Wang X Chupp GL Montgomery RR. Profiling Cellular Heterogeneity in Asthma With Single Cell Multiparameter CyTOF. J Leukoc Biol (2020) 108:1555–64. 10.1002/JLB.5MA0720-770RR PMC8087109 32911570

[50] Goswami R Blazquez AB Kosoy R Rahman A Nowak-Wegrzyn A Berin MC. Systemic Innate Immune Activation in Food Protein-Induced Enterocolitis Syndrome. J Allergy Clin Immunol (2017) 139:1885–96.e9. 10.1016/j.jaci.2016.12.971 28192147 PMC5461215

[51] Mehr S Lee E Hsu P Anderson D de Jong E Bosco A. Innate Immune Activation Occurs in Acute Food Protein-Induced Enterocolitis Syndrome Reactions. J Allergy Clin Immunol (2019) 144:600–2.e2. 10.1016/j.jaci.2019.04.021 31059725

[52] Berin MC. Advances in Understanding Immune Mechanisms of Food Protein-Induced Enterocolitis Syndrome. Ann Allergy Asthma Immunol (2021) 126:478–81. 10.1016/j.anai.2021.01.033 33548465

[53] Macosko EZ Basu A Satija R Nemesh J Shekhar K Goldman M. Highly Parallel Genome-Wide Expression Profiling of Individual Cells Using Nanoliter Droplets. Cell (2015) 161:1202–14. 10.1016/j.cell.2015.05.002 PMC4481139 26000488

[54] He H Suryawanshi H Morozov P Gay-Mimbrera J Del Duca E Kim HJ. Single-Cell Transcriptome Analysis of Human Skin Identifies Novel Fibroblast Subpopulation and Enrichment of Immune Subsets in Atopic Dermatitis. J Allergy Clin Immunol (2020) 145:1615–28. 10.1016/j.jaci.2020.01.042 32035984

[55] Czarnowicki T He H Krueger JG Guttman-Yassky E. Atopic Dermatitis Endotypes and Implications for Targeted Therapeutics. J Allergy Clin Immunol (2019) 143:1–11. 10.1016/j.jaci.2018.10.032 30612663

[56] Wen T Aronow BJ Rochman Y Rochman M Kc K Dexheimer PJ. Single-Cell RNA Sequencing Identifies Inflammatory Tissue T Cells in Eosinophilic Esophagitis. J Clin Invest (2019) 129:2014–28. 10.1172/JCI125917 PMC6486341 30958799

[57] Azouz NP Klingler AM Pathre P Besse JA Baruch-Morgenstern NB Ballaban AY. Functional Role of Kallikrein 5 and Proteinase-Activated Receptor 2 in Eosinophilic Esophagitis. Sci Transl Med (2020) 12:7773–86. 10.1126/scitranslmed.aaz7773 PMC7350155 32461336

[58] Dunn JLM Shoda T Caldwell JM Wen T Aceves SS Collins MH. Esophageal Type 2 Cytokine Expression Heterogeneity in Eosinophilic Esophagitis in a Multisite Cohort. J Allergy Clin Immunol (2020) 145:1629–40.e4. 10.1016/j.jaci.2020.01.051 32197970 PMC7309223

[59] Ruffner MA Hu A Dilollo J Benocek K Shows D Gluck M. Conserved IFN Signature Between Adult and Pediatric Eosinophilic Esophagitis. J Immunol (2021) 206:1361–71. 10.4049/jimmunol.2000973 PMC7946729 33558373

[60] Smith JG Gerszten RE. Emerging Affinity-Based Proteomic Technologies for Large-Scale Plasma Profiling in Cardiovascular Disease. Circulation (2017) 135:1651–64. 10.1161/CIRCULATIONAHA.116.025446 PMC5555416 28438806

[61] Pavel AB Zhou L Diaz A Ungar B Dan J He H. The Proteomic Skin Profile of Moderate-to-Severe Atopic Dermatitis Patients Shows an Inflammatory Signature. J Am Acad Dermatol (2020) 82:690–9. 10.1016/j.jaad.2019.10.039 31669080

[62] Leonard A Wang J Yu L Liu H Estrada Y Greenlees L. Atopic Dermatitis Endotypes Based on Allergen Sensitization, Reactivity to Staphylococcus Aureus Antigens, and Underlying Systemic Inflammation. J Allergy Clin Immunol Pract (2020) 8:236–47.e3. 10.1016/j.jaip.2019.08.013 31430591

[63] Rossios C Pavlidis S Hoda U Kuo CH Wiegman C Russell K. Sputum Transcriptomics Reveal Upregulation of IL-1 Receptor Family Members in Patients With Severe Asthma. J Allergy Clin Immunol (2018) 141:560–70. 10.1016/j.jaci.2017.02.045 28528200

[64] Grapov D Fahrmann J Wanichthanarak K Khoomrung S. Rise of Deep Learning for Genomic, Proteomic, and Metabolomic Data Integration in Precision Medicine. OMICS (2018) 22:630–6. 10.1089/omi.2018.0097 PMC6207407 30124358

[65] LeCun Y Bengio Y Hinton G. Deep Learning. Nature (2015) 521:436–44. 10.1038/nature14539 26017442

[66] Min S Lee B Yoon S. Deep Learning in Bioinformatics. Brief Bioinform (2017) 18:851–69. 10.1093/bib/bbw068 27473064

[67] Wild CP. Complementing the Genome With an “Exposome”: The Outstanding Challenge of Environmental Exposure Measurement in Molecular Epidemiology. Cancer Epidemiol Biomarkers Prev (2005) 14:1847–50. 10.1158/1055-9965.EPI-05-0456 16103423

[68] Wild CP. The Exposome: From Concept to Utility. Int J Epidemiol (2012) 41:24–32. 10.1093/ije/dyr236 22296988

[69] Dagnino S Macherone A. Unravelling the Exposome: Conclusions and Thoughts for the Future. Unraveling the Exposome: A Practical View. 1st ed. Cham: Springer International Publishing: Imprint: Springer (2019). p. 425–37.

[70] Siroux V Agier L Slama R. The Exposome Concept: A Challenge and a Potential Driver for Environmental Health Research. Eur Respir Rev (2016) 25:124–9. 10.1183/16000617.0034-2016 PMC9487242 27246588

[71] Vermeulen R Schymanski EL Barabasi AL Miller GW. The Exposome and Health: Where Chemistry Meets Biology. Science (2020) 367:392–6. 10.1126/science.aay3164 PMC7227413 31974245

[72] Martin-Sanchez F Bellazzi R Casella V Dixon W Lopez-Campos G Peek N. Progress in Characterizing the Human Exposome: A Key Step for Precision Medicine. Yearb Med Inform (2020) 29:115–20. 10.1055/s-0040-1701975 PMC7442499 32303099

[73] Wright RO. Exposomics and Precision Medicine: Everything That Rises Must Converge. [Online]. New York: Institute for Exposomic Research, Icahn School of Medicine at Mount Sinai (2019). Available: https://ukcares.med.uky.edu/sites/default/files/Expsome_PMI_Talk_2019_Wright_final%20%28EMPH_038%27s%20conflicted%20copy%202019-08-07%29.pdf [Accessed May 15 2021].

[74] Stefanovic N Flohr C Irvine AD. The Exposome in Atopic Dermatitis. Allergy (2020) 75:63–74. 10.1111/all.13946 31194890 PMC7003958

[75] Guillien A Cadiou S Slama R Siroux V. The Exposome Approach to Decipher the Role of Multiple Environmental and Lifestyle Determinants in Asthma. Int J Environ Res Public Health (2021) 18:1138–51. 10.3390/ijerph18031138 PMC7908097 33525356

[76] Burbank AJ Sood AK Kesic MJ Peden DB Hernandez ML. Environmental Determinants of Allergy and Asthma in Early Life. J Allergy Clin Immunol (2017) 140:1–12. 10.1016/j.jaci.2017.05.010 28673399 PMC5675123

[77] Alkotob SS Cannedy C Harter K Movassagh H Paudel B Prunicki M. Advances and Novel Developments in Environmental Influences on the Development of Atopic Diseases. Allergy (2020) 75:3077–86. 10.1111/all.14624 PMC12087019 33037680

[78] Baluch N Gallant M Ellis AK. Exposomal Research in the Context of Birth Cohorts: What Have They Taught Us? Ann Allergy Asthma Immunol (2020) 125:639–45. 10.1016/j.anai.2020.09.006 32927048

[79] Doherty BT Koelmel JP Lin EZ Romano ME Godri Pollitt KJ. Use of Exposomic Methods Incorporating Sensors in Environmental Epidemiology. Curr Environ Health Rep (2021) 8:34–41. 10.1007/s40572-021-00306-8 33569731

[80] Bui AAT Hosseini A Rocchio R Jacobs N Ross MK Okelo S. Biomedical REAl-Time Health Evaluation (BREATHE): Toward an Mhealth Informatics Platform. JAMIA Open (2020) 3:190–200. 10.1093/jamiaopen/ooaa011 32734159 PMC7382637

[81] Johansson H Mersha TB Brandt EB Khurana Hershey GK. Interactions Between Environmental Pollutants and Genetic Susceptibility in Asthma Risk. Curr Opin Immunol (2019) 60:156–62. 10.1016/j.coi.2019.07.010 PMC6800636 31470287

[82] Mersha TB Beck AF. The Social, Economic, Political, and Genetic Value of Race and Ethnicity in 2020. Hum Genomics (2020) 14:37. 10.1186/s40246-020-00292-2 33059745 PMC7558251

[83] Raffield LM Dang H Pratte KA Jacobson S Gillenwater LA Ampleford E. Comparison of Proteomic Assessment Methods in Multiple Cohort Studies. Proteomics (2020) 20:e1900278. 10.1002/pmic.201900278 32386347 PMC7425176

[84] Luecken MD Theis FJ. Current Best Practices in Single-Cell RNA-Seq Analysis: A Tutorial. Mol Syst Biol (2019) 15:e8746. 10.15252/msb.20188746 31217225 PMC6582955

